# Localization of ^99m^Tc-GRP Analogs in GRPR-Expressing Tumors: Effects of Peptide Length and Neprilysin Inhibition on Biological Responses

**DOI:** 10.3390/ph12010042

**Published:** 2019-03-20

**Authors:** Aikaterini Kaloudi, Emmanouil Lymperis, Panagiotis Kanellopoulos, Beatrice Waser, Marion de Jong, Eric P. Krenning, Jean Claude Reubi, Berthold A. Nock, Theodosia Maina

**Affiliations:** 1 Molecular Radiopharmacy, INRASTES, NCSR “Demokritos”, 15310 Athens, Greece; katerinakaloudi@yahoo.gr (A.K.); mlymperis@hotmail.com (E.L.); kanelospan@gmail.com (P.K.); nock_berthold.a@hotmail.com (B.A.N.); 2 Cell Biology and Experimental Cancer Research, Institute of Pathology, University of Berne, CH-3010 Berne, Switzerland; waserpatho@rubigen.ch (B.W.); jean.reubi@pathology.unibe.ch (J.C.R.); 3 Department of Radiology & Nuclear Medicine Erasmus MC, 3015 CN Rotterdam, The Netherlands; m.hendriks-dejong@erasmusmc.nl; 4 Cytrotron Rotterdam BV, Erasmus MC, 3015 CN Rotterdam, The Netherlands; erickrenning@gmail.com

**Keywords:** bombesin, gastrin-releasing peptide, gastrin-releasing peptide receptor, tumor targeting, ^99m^Tc-radioligand, metabolic stability, neprilysin-inhibition, phosphoramidon

## Abstract

The overexpression of gastrin-releasing peptide receptors (GRPRs) in frequently occurring human tumors has provided the opportunity to use bombesin (BBN) analogs as radionuclide carriers to cancer sites for diagnostic and therapeutic purposes. We have been alternatively exploring human GRP motifs of higher GRPR selectivity compared to frog BBN sequences aiming to improve pharmacokinetic profiles. In the present study, we compared two differently truncated human endogenous GRP motifs: GRP(14–27) and GRP(18–27). An acyclic tetraamine was coupled at the N-terminus to allow for stable binding of the SPECT radionuclide ^99m^Tc. Their biological profiles were compared in PC-3 cells and in mice without or with coinjection of phosphoramidon (PA) to induce transient neprilysin (NEP) inhibition in vivo. The two ^99m^Tc-N_4_-GRP(14/18–27) radioligands displayed similar biological behavior in mice. Coinjection of PA exerted a profound effect on in vivo stability and translated into notably improved radiolabel localization in PC-3 experimental tumors. Hence, this study has shown that promising ^99m^Tc-radiotracers for SPECT imaging may indeed derive from human GRP sequences. Radiotracer bioavailability was found to be of major significance. It could be improved during in situ NEP inhibition resulting in drastically enhanced uptake in GRPR-expressing lesions.

## 1. Introduction

Gastrin-releasing peptide receptors (GRPRs) are overexpressed in several human malignancies such as prostate cancer, mammary carcinoma, and lung cancer [1,2,3,4,5,6,7,8,9,10]. Consequently, they have attracted considerable attention as potential biomolecular targets for diagnosis and therapy with radionuclide carriers directed to GRPR-positive cancer lesions [11,12]. Originally, the frog tetradecapeptide bombesin (BBN, Pyr-Gln-Arg-Tyr-Gly-Asn-Gln-Trp-Ala-Val-Gly-His-Leu-Met-NH_2_) and its truncated C-terminal octapeptide fragment BBN(7–14) have served as motifs for the development of GRPR-targeting radioligands. However, BBN-like analogs bind with comparable affinity not only to GRPR (BB_2_R), but also to the neuromedin B (NMBR, BB_1_R), another member of the three mammalian bombesin receptor subtypes [1,2]. The above two subtypes are pharmacologically distinguished by their selectivity for different endogenous human homologs of amphibian BBN. Thus, the 27-mer GRP (H-Val-Pro-Leu-Pro-Ala-Gly-Gly-Gly-Thr- Val-Leu-Thr-Lys-Met-Tyr-Pro-Arg-Gly-Asn-His-Trp-Ala-Val-Gly-His-Leu-Met-NH_2_), the 14-mer GRP(14–27), and the C-terminal decapeptide GRP(18–27) fragments strongly bind to the GRPR, whereas neuromedin B (NMB, H-Gly-Asn-Leu-Trp-Ala-Thr-Gly-His-Phe-Met-NH_2_) exhibits high affinity for the NMBR [13]. The two GRPR and NMBR subtypes are physiologically expressed in the human brain and the gut, especially in stomach, pancreas, and gastrointestinal tract, and they are also implicated in cancer [14,15,16]. It is reasonable to assume, that radiolabeled BBN agonists of poor GRPR selectivity will show increased levels of background radioactivity by virtue of their binding to both GRPR and NMBR populations distributed in the body, especially in the abdomen. Furthermore, additive GRPR- and NMBR-mediated effects in the gastrointestinal tract, such as abdominal smooth muscle contraction and stimulation of gastrointestinal hormone secretion, are to be expected after intravenous injection of BBN-like agonist radioligands [17,18,19,20,21,22].

In contrast to amphibian BBN-like motifs, the respective human homologs have surprisingly remained unexploited as radionuclide carriers for targeting GRPR-positive cancer [13]. Motivated by this gap in the inventory of GRPR-directed radioligands we have expanded our research efforts to native human GRP sequences in order to explore their applicability in GRPR-targeted tumor diagnosis and therapy. First, we introduced a small library of tetraamine derivatized GRP(18–27) analogs labeled with the SPECT radionuclide ^99m^Tc [23,24]. Compared to previously reported ^99m^Tc-radiopeptides, which are based on the full-length BBN or its truncated BBN(7–14) octapeptide fragment [25], the ^99m^Tc-N_4_-GRP(18–27) showed high GRPR selectivity and superior in vivo characteristics in tumor-bearing mice, such as faster renal clearance and improved tumor to background ratios. On the other hand, single or double amino acid substitutions in the decapeptide backbone exerted pronounced effects on several biological properties, eventually affecting tumor targeting capabilities and pharmacokinetics. In a following study, a series of differently truncated GRP sequences were coupled to the universal chelator DOTA (1,4,7,10- tetraazacyclododecane-1,4,7,10-tetraacetic acid) and labeled with ^111^In. Receptor affinity, internalization efficiency and tumor uptake of these analogs were favored both by longer peptide chain and by the presence of basic amino acids Lys^13^ and Arg^17^ in the native GRP sequence [26].

**Figure 1:**
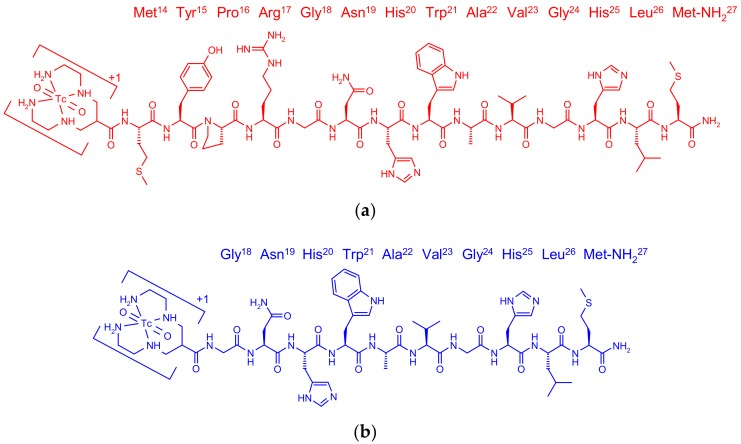
Chemical structure of (**a**) ^99m^Tc-N_4_-GRP(14–27) (red) and (**b**) ^99m^Tc-N_4_-GRP(18–27) (blue).

Following this line of research, we herein introduced ^99m^Tc-N_4_-GRP(14–27) and compared its biological profile in PC-3 cells and mice models with ^99m^Tc-N_4_-GRP(18–27) (Figure 1). It should be noted that basic positions Lys^13^ and Arg^17^ in the native GRP sequence are now occupied by the positively charged N_4_^+x^/[^99m^Tc(O)_2_(N_4_)]^+1^-moiety and not by the negatively charged DOTA. This arrangement allows for comparisons with the DOTA-derivatized analogs and further studying the influence of positive/negative charges in 13 and 17 positions of the GRP chain [26]. Next, the selectivity of N_4_-GRP(14–27) for each of the three mammalian bombesin receptor subtypes was investigated applying receptor autoradiography in human excised biopsy samples, expressing one of the GRPR, NMBR, and bombesin subtype 3 (BB_3_R) receptors. Finally, the impact of in vivo stability of ^99m^Tc-N_4_-GRP(14–27) and ^99m^Tc-N_4_-GRP(18–27) on tumor targeting and pharmacokinetics was compared in mice. The role of neprilysin (NEP) [27] on the in vivo degradation of the two human GRP-based sequences was monitored by HPLC analysis of blood samples collected without or with coinjection of the NEP-inhibitor phosphoramidon (PA) [28,29], as previously described for BBN-like radioligands [30,31,32,33,34]. The enhancement of radiotracer localization in experimental GRPR-positive PC-3 tumors in mice during transient NEP inhibition induced by PA was assessed.

## 2. Results

### 2.1. Peptides and Radioligands

**Figure 2:**
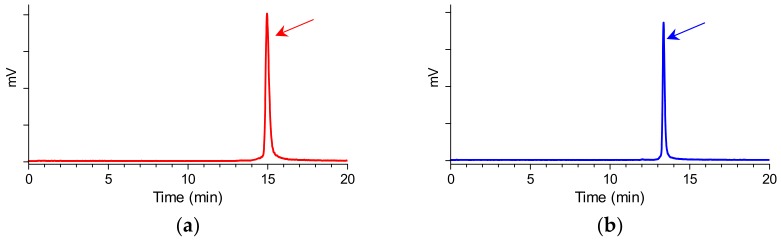
Representative radiochromatograms of the radiolabeling reaction mixture of (**a**) ^99m^Tc-N_4_-GRP(14–27) (red) and (**b**) ^99m^Tc-N_4_-GRP(18–27) (blue), confirming the quantitative formation of high purity radioligands at *t_R_* = 14.9 min and *t_R_* = 13.3 min, respectively (HPLC system 1).

The bifunctional acyclic tetraamine chelator (6-(carboxy)-1,4,8,11-tetraazaundecane) was covalently coupled by its carboxy functionality at the N-terminal Met^14^ of GRP(14–27) or the Gly^18^ of GRP(18–27) via an amide bond [24], generating two different length GRP analogs amenable for labeling with the preeminent SPECT radionuclide ^99m^Tc. Labeling was typically proceeded by a brief incubation with ^99m^TcO_4_^−^ generator eluate, SnCl_2_ as reducing agent, and citrate anions as transfer ligand in alkaline pH at ambient temperature at molar activities of 20 to 40 MBq ^99m^Tc/nmol peptide. Quality control of the radiolabeled products combined HPLC and ITLC analysis. The total radiochemical impurities, comprising ^99m^TcO_4_^−^, [^99m^Tc] citrate, and ^99m^TcO_2_ × H_2_O, did not exceed 2%, while a single radiopeptide species was detected by RP-HPLC. In view of labeling yields >98% and >99% radiochemical purity of the resultant ^99m^Tc-N_4_-GRP(14–27) and ^99m^Tc-N_4_-GRP(18–27), the radioligands were used without further purification in all subsequent experiments. Representative radiochromatograms of HPLC analysis of ^99m^Tc-N_4_-GRP(14–27) and ^99m^Tc-N_4_-GRP(18–27) are included in Figure 2a,b, respectively.

### 2.2. In Vitro Assays

#### 2.2.1. Receptor Autoradiography in Human Tumor Samples

The selective affinities of N_4_-GRP(14–27) for each of the three bombesin receptor subtypes found in mammals were studied during in vitro competition binding assays against the universal radioligand ^125^I-[DTyr^6^,*β*Ala^11^,Phe^13^,Nle^14^]BBN(6–14) [6]. Receptor autoradiography was applied in cryostat sections of well characterized human cancers, preferentially expressing one of the subtypes. As summarized in Table 1, N_4_-GRP(14–27) showed high affinity for the GRPR expressed in resected prostate carcinoma specimens (IC_50_ = 4.2 ± 1.0 nM, *n* = 3, vs. IC_50_ = 2.4 ± 1.0 nM, *n* = 3 for N_4_-GRP(18–27) [23]), very low affinity for NMBR present in ileal carcinoid biopsy samples (IC_50_ = 72 ± 7.6 nM, *n* = 3, vs. IC_50_ = 106 ± 13 nM; *n* = 2 for N_4_-GRP(18–27) [23]), and no affinity for the BB_3_R expressed in bronchial carcinoid samples (IC_50_ > 1000 nM, *n* = 3, identical to N_4_-GRP(18–27) [23]). Thus, N_4_-GRP(14–27) similarly to N_4_-GRP(18–27), displayed good selectivity for the GRPR. Hence, the GRP-based analogs turned out to be more GRPR-preferring compared to BBN-based radioligands, like Demobesin 3 (N_4_-[Pro^1^,Tyr^4^]BBN) [25] or [DTyr^6^,*β*Ala^11^,Phe^13^,Nle^14^]BBN(6–14) (Table 1).

#### 2.2.2. Binding Affinity for the Human GRPR

**Figure 3:**
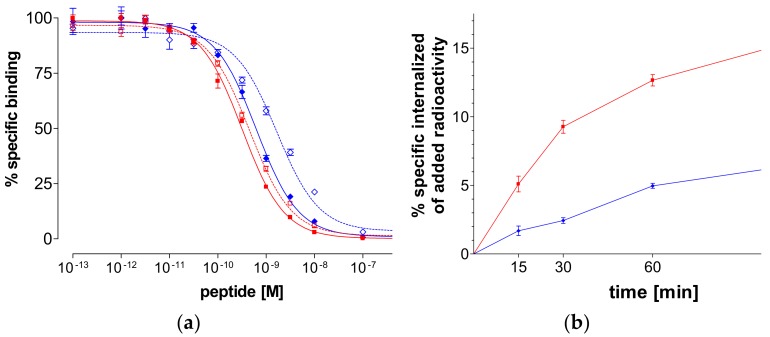
(**a**) [^125^I-Tyr^4^]BBN displacement curves from gastrin-releasing peptide receptor (GRPR)-sites on PC-3 cells after 1-h incubation at 22 °C by N_4_-GRP(14–27) (red solid line—IC_50_ 0.32±0.03 nM), GRP(14–27) (red dashed line—IC_50_ 0.45 ± 0.02 nM), N_4_-GRP(18–27) (blue solid line—IC_50_ 0.63±0.06 nM) and GRP(18–27) (blue dashed line—IC_50_ 1.66 ± 0.20 nM). (**b**) GRPR-specific internalization of ^99m^Tc-N_4_-GRP(14–27) (red solid line) and ^99m^Tc-N_4_-GRP(18–27) (blue solid line) in PC-3 cells during incubation at 37 °C at 15, 30, 60, and 120 min. Results represent average of cell internalized activity ± sd, *n* = 3; data is corrected for nonspecific internalization in the presence of 1 µM [Tyr^4^]BBN.

As shown in Figure 3a, N_4_-GRP(14–27) and N_4_-GRP(18–27) as well as the respective GRP(14–27) and GRP(18–27) parent peptide references displaced [^125^I-Tyr^4^]BBN from GRPR-sites on PC-3 cell membranes in a monophasic and dose-dependent manner. The respective half-maximal inhibitory concentration (IC_50_) values differed, yielding the following rank of decreasing receptor affinity: N_4_-GRP(14–27) (IC_50_ 0.32 ± 0.03 nM) > GRP(14–27) (IC_50_ 0.45 ± 0.02 nM) > N_4_-GRP(18–27) (IC_50_ 0.63 ± 0.06 nM) > GRP(18–27) (IC_50_ 1.66 ± 0.20 nM). We observe that the longer-chain peptides consistently showed higher binding affinity to GRPR than their shorter chain counterparts. Moreover, coupling of the positively charged acyclic tetraamine unit in the N-terminus of parent GRP(14/18–27) references improved the affinity of resulting analogs to the GRPR, as previously reported for similarly modified peptide analogs [35].

#### 2.2.3. Internalization of ^99m^Tc-N_4_-GRP(14–27) and ^99m^Tc-N_4_-GRP(18–27) in PC-3 Cells

During incubation at 37 °C in PC-3 cells, both ^99m^Tc-N_4_-GRP(14–27) and ^99m^Tc-N_4_-GRP(18–27) were taken up by the cells via a GRPR-mediated process, as demonstrated by the lack of internalization observed in the presence of excess [Tyr^4^]BBN. In both cases the bulk of cell-associated radioactivity was found in the cells with ^99m^Tc-N_4_-GRP(14–27) internalizing much faster in PC-3 cells compared to ^99m^Tc-N_4_-GRP(18–27) at all time intervals (Figure 3b). For example, at 1 h, 12.7 ± 0.7% of total added ^99m^Tc-N_4_-GRP(14–27) specifically internalized in the cells vs. 5.0 ± 0.3% of ^99m^Tc-N_4_-GRP(18–27), whereas at 2 h these values increased to 19.5 ± 1.4% and 6.9 ± 1.5%, respectively.

### 2.3. In Vivo Comparison of ^99m^Tc-N_4_-GRP(14–27) and ^99m^Tc-N_4_-GRP(18–27)

#### 2.3.1. Stability of ^99m^Tc-N_4_-GRP(14–27) and ^99m^Tc-N_4_-GRP(18–27) in Mice

**Figure 4:**
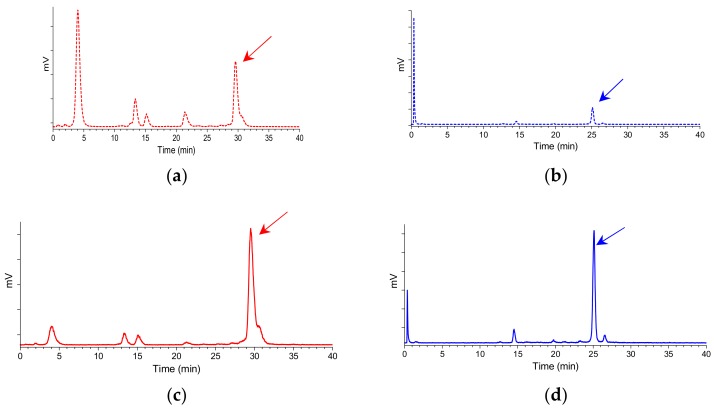
Representative radiochromatograms of HPLC analysis of mouse blood samples collected 5 min pi of (**a**) ^99m^Tc-N_4_-GRP(14–27) (25.2% intact radiotracer; red dashed line) or (**b**) ^99m^Tc-N_4_-GRP(18–27) (31.8% intact radiotracer; blue dashed line) without PA coinjection; the respective radiochromatograms of (**c**) ^99m^Tc-N_4_-GRP(14–27) (63.1% intact radiotracer; *t*_R_ = 29.6 min; red solid line) or (**d**) ^99m^Tc-N_4_-GRP(18–27) (68.1% intact radiotracer; *t*_R_ = 25.1 min; blue solid line) with PA coinjection are also included; the *t*_R_ of parent radiopeptide was determined by coinjection with the respective radioligand sample in the column (HPLC system 2) and is indicated here by the arrow.

The two ^99m^Tc-N_4_-GRP(14–27) and ^99m^Tc-N_4_-GRP(18–27) radiotracers exhibited distinct resistance to degrading proteases after injection in mice. As revealed by HPLC analysis of blood samples collected at 5 min postinjection (pi), ^99m^Tc-N_4_-GRP(14–27) was found less stable (20.1 ± 4.5% intact, *n* = 3) than the shorter chain ^99m^Tc-N_4_-GRP(18–27) (31.0 ± 0.9% intact, *n* = 3). Representative radiochromatograms are shown in Figure 4a,b, respectively.

It should be noted that coinjection of the NEP-inhibitor PA remarkably enhanced the in vivo stability of ^99m^Tc-N_4_-GRP(14–27) (66.5 ± 4.8% intact, *n* = 3) and ^99m^Tc-N_4_-GRP(18–27) (70.8 ± 5.4% intact, *n* = 3) in the circulation, revealing NEP as a major degrading protease for both radiotracers in mice. Representative radiochromatograms are included in Figure 4c,d, respectively.

#### 2.3.2. Biodistribution in PC-3 Xenograft-Bearing Mice

The biodistribution of ^99m^Tc-N_4_-GRP(14–27) and ^99m^Tc-N_4_-GRP(18–27) was studied in severe combined immune deficiency (SCID) mice bearing human PC-3 xenografts expressing the human GRPR. Subcutaneous tumors of suitable size developed in the flanks of mice about four weeks after inoculation of a suspension of prostate adenocarcinoma PC-3 cells and biodistribution was conducted.

Cumulative biodistribution results for ^99m^Tc-N_4_-GRP(14–27) at the 1-, 4-, and 24-h pi intervals are summarized in Table 2, and are expressed as mean % injected dose per gram (%ID/g) values ± sd, *n* = 4. The radiotracer washed rapidly from the blood and the background tissues predominantly via the kidneys and the urinary system. High uptake was observed in the PC-3 tumor at 1-h pi (10.20 ± 0.72%ID/g) that remained at comparably high levels at 4-h pi (8.41 ± 4.16%ID/g; *p* > 0.05), declining by ~50% at 24-h pi (4.50 ± 0.69%ID/g). Tumor uptake at 4-h pi was significantly lower in the animals treated with excess [Tyr^4^]BBN (0.62 ± 0.24%ID/g; *p* < 0.001), suggestive of a GRPR-mediated process. Likewise, ^99m^Tc-N_4_-GRP(14–27) highly localized in the GRPR-rich mouse pancreas via a GRPR-specific process, as demonstrated by the lack of pancreatic uptake during GRPR-blockade by coinjection of excess [Tyr^4^]BBN (35.24 ± 4.70%ID/g vs. 0.83 ± 0.24%ID/g in block; *p* < 0.001).

Comparative biodistribution results for ^99m^Tc-N_4_-GRP(14–27) and ^99m^Tc-N_4_-GRP(18–27) at the 4-h pi interval are included in Table 3. Data from additional 4-h pi animal groups coinjected with the NEP-inhibitor PA (300 µg) is also included in the Table. The radiotracers displayed similar tissue distribution patterns. The observed higher tumor and pancreatic uptake of ^99m^Tc-N_4_-GRP(14–27) did not however differ significantly to that of ^99m^Tc-N_4_-GRP(18–27) (*p* > 0.05) [24]. Treatment with PA induced a drastic increase in tumor values for both radiotracers with ^99m^Tc-N_4_-GRP(14–27) showing superior tumor values than the shorter chain ^99m^Tc-N_4_-GRP(18–27) (38.19 ± 4.79%ID/g vs. 28.37 ± 8.05%ID/g, respectively; *p* < 0.01). Pancreatic values increased by >3-fold for both radiotracers as well. Thus, in agreement with previous studies on BBN-based analogs [31,32,33,34], NEP inhibition likewise resulted in significant stabilization of GRP(14/18–27)-based radioligands in peripheral mouse blood and notable improvement of localization in GRPR-expressing lesions in mice.

## 3. Discussion

A considerable number of radiolabeled analogs of frog BBN have been developed for potential application in the diagnosis and therapy of GRPR-expressing tumors in *Homo sapiens* [11,12]. This pursuit is based on the overexpression of GRPRs on the surface of malignant cells serving as easily accessible biomolecular targets on cancer lesions [3,4,5,6,7,8,9,10]. Joining this effort, we have previously introduced a series of BBN-like analogs, generated by covalently coupling of an acyclic tetraamine at the N-terminus, Demobesin 3–6 [25]. Like native BBN, these peptide ligands displayed indistinguishable binding affinities for the human bombesin receptor subtypes GRPR and NMBR, but no affinity for the BB_3_R. The respective radiotracers [^99m^Tc] Demobesin 3–6 specifically localized in GRPR-expressing human PC-3 xenografts in mice. Moreover, one of the analogs, [^99m^Tc] Demobesin 4, was able to visualize malignant lesions in a small number of prostate cancer patients with SPECT/CT [36].

We have recently expanded our search toward radioligands based on human endogenous GRP sequences [23,24,26]. The latter are reported for higher GRPR selectivity compared to their frog homolog BBN [13]. This approach has surprisingly remained unexplored up to now, although it offers two major advantages. First, radioactivity levels in abdominal tissues physiologically expressing both GRPR and NMBR subtypes would favorably decrease when GRPR-selective radioligands are used [14,15,16]. Second, injection of GRPR-selective agonists will not activate the NMBR subtype populations in the gut and is hence associated with less adverse effects [17,18,19,20,21,22]. Promising GRPR-selective radioligands based on receptor antagonists have been developed and evaluated in animals and in human, showing excellent tumor targeting and pharmacokinetic profiles [37,38]. Yet, the suitability and efficacy of noninternalizing GRPR antagonists for therapy with low-range beta, alpha, or Auger emitters of high LET has not been established thus far [39,40]. Therefore, the study of radiolabeled GRPR-selective agonists is warranted and may provide an alternative platform for the development of radioligands exhibiting new combinations of biological features particularly suited for cancer theranostics.

As a part of this effort we herein introduced two analogs of endogenous truncated fragments of human GRP carrying an acyclic tetraamine at their terminus, one based on the tetradecapeptide GRP(14–27), and the other on a shorter decapeptide GRP(18–27) chain identical to human neuromedin C (NMC) (Figure 1). As revealed during receptor autoradiography studies in excised biopsy samples of well characterized human tumors expressing each of the GRPR, NMBR, and BB_3_R subtypes using the universal ^125^I-[DTyr^6^,*β*Ala^11^,Phe^13^,Nle^14^]BBN(6–14) radioligand, both N_4_-GRP(14–27) and N_4_-GRP(18–27) analogs showed good selectivity for the GRPR subtype (Table 1). This finding is in agreement with previous reports documenting the preference of human GRP and its C-terminal native fragments for GRPR [13]. In contrast, the tetraamine derivatized BBN-like analogs Demobesin 3–6 do not distinguish between GRPR and NMBR, instead behaving like the native frog BBN [25]. The His^20^ of GRP corresponding to Gln^7^ of BBN seems to have significant impact on subtype selectivity, followed by the Met^14^-Tyr^13^-Pro^12^-Arg^11^-tetrapeptide in GRP(14–27) corresponding to the Pyr^1^-Gln^2^-Arg^3^-Leu^4^-counterpart in native BBN.

Both N_4_-GRP(14–27) and N_4_-GRP(18–27) analogs displayed sub-nM affinities for the GRPR during competition assays against [^125^I-Tyr^4^]BBN in PC-3 cell membranes, which were found to be increased compared to unmodified GRP(14/18–27) lead structures (Figure 3a). It is interesting to note that the presence of the positively charged N_4_^+x^-moiety at positions 13 and 17 of the native GRP chain occupy the two basic amino acids Lys^13^ and Arg^17^. When these positions were taken up by negatively charged DOTA instead, a drastic drop in binding affinity was observed (IC50 DOTA-GRP(14–27) = 6.6 ± 0.9 nM and IC50 DOTA-GRP(18–27) = 112 ± 16 nM) [26]. In all cases, longer chain GRP(14–27) analogs consistently displayed higher GRPR-affinity compared to their C-terminal decapeptide counterparts. In agreement with this observation, the internalization rate of ^99m^Tc-N_4_-GRP(14–27) in PC-3 cells was clearly superior to that of ^99m^Tc-N_4_-GRP(18–27) (Figure 3b).

A crucial feature for efficient tumor targeting is metabolic stability that will ensure sufficient radioligand supply to tumor sites after injection in the living organism [31]. Recent studies have revealed the significance of in vivo stability assessment of BBN-like radiotracers vs. in vitro determinations in serum or tissue homogenate incubates to more accurately predict the actual protease(s) encountered by the radioligand after entering the circulation. These studies have demonstrated NEP as a major degrading protease of BBN and its radiolabeled analogs [41,42]. In a recently proposed approach, NEP-inhibitors, like PA [28,29], coinjected with the BBN-based radioligand drastically increased metabolic stability leading to notable enhancement of tumor uptake in animal models [31,32,33,34]. Following this rationale, we have tested the in vivo stability of the two ^99m^Tc-N_4_-GRP(14–27) and ^99m^Tc-N_4_-GRP(18–27) radiotracers in peripheral mouse blood collected 5 min pi by radioanalytical HPLC (Figure 4). The longer chain ^99m^Tc-N_4_-GRP(14–27) displayed somewhat poorer stability (20.1 ± 4.5% intact, *n* = 3) compared to shorter chain ^99m^Tc-N_4_-GRP(18–27) (31.0 ± 0.9% intact, *n* = 3). Thus, the presence of additional amide bonds in the longer peptide chain offered further degradation sites for attacking proteases. It is interesting to note, that coinjection of PA drastically increased the stability of both analogs in the circulation, indicating NEP as a major protease in the catabolism of GRP sequences as well.

Aiming to explore the effects of peptide chain as well as NEP inhibition on the pharmacokinetics and tumor targeting capabilities of the two tracers, we have conducted biodistribution experiments in immunosuppressed mice bearing human GRPR-expressing xenografts. Interestingly, the biodistribution patterns of the two radiotracers did not clearly differ (Table 3). Compared to the previously reported ^111^In-DOTA analogs [26], ^99m^Tc-N_4_-GRP(14–27) showed higher tumor uptake. Comparable in vivo tumor targeting was obtained only by ^111^In-DOTA-GRP(13–27), carrying basic Lys at position 13. However, kidney clearance was notably faster for the ^99m^Tc-radiotracer (4.83 ± 2.12%/g at 4 h pi) than for the ^111^In-DOTA-Lys^13^-analog (46.02 ± 3.73%/g at 4 h pi) [26]. Treatment of mice with PA exerted a profound effect on the in vivo profile of both ^99m^Tc-N_4_-GRP(14–27) and ^99m^Tc-N_4_-GRP(18–27) (Table 2), is in agreement with previous findings for other BBN-like radioligands. The longer chain ^99m^Tc-N_4_-GRP(14–27) exhibited the highest uptake in the experimental tumor, as a combined result of higher internalization rate and pronounced in vivo stabilization induced by PA.

The present study has shown that GRP motifs of different chain length may provide new opportunities for the development of promising GRPR-selective radioligands. The latter may prove to be a useful asset in the arsenal of anti-GRPR radioactive drugs, by internalizing in cancer cells while evading binding to and activation of bombesin receptor subtypes other than the GRPR in the gut. Furthermore, it has shown the impact of in vivo metabolic stability in maximizing tumor localization. Structural modifications in peptide lead-structures to improve the stability of radiopeptides, such as amino acid replacements, cyclization, or changes of cleavable peptide bonds, may deteriorate other important biological features, as for example receptor affinity, cell uptake, tumor targeting, and overall pharmacokinetics. On the other hand, transient in situ inhibition of NEP represents a smart method to accomplish this goal and warrants further efforts for translation in the clinic. Despite challenges related to biosafety, regulatory and financial hurdles, once established in a proof-of-principal study, this strategy is expected to boost the development and clinical application of other NEP-catabolized radioligands, considerably saving costs and resources [31,32,33,34].

## 4. Materials and Methods

### 4.1. Peptides and Reagents

The N_4_-GRP(14–27) and N_4_-GRP(18–27) peptide conjugates were synthesized on the solid support following a published method [24] and were provided by PiChem (Graz, Austria). The [Tyr^4^]BBN (Tyr^4^-bombesin, Pyr-Gln-Arg-Tyr-Gly-Asn-Gln-Trp-Ala-Val-Gly-His-Leu-Met-NH_2_) and GRP(14–27) (H-Met-Tyr-Pro-Arg-Gly-Asn-His-Trp-Ala-Val-Gly-His-Leu-Met-NH_2_) references were purchased from PSL GmbH (Heidelberg, Germany), whereas GRP(18–27) (H-Gly-Asn-His-Trp-Ala- Val-Gly-His-Leu-Met-NH_2_) was provided by PeptaNova GmbH (Sandhousen, Germany). Phosphoramidon disodium dehydrate (N-(α-rhamnopyranosyloxyhydro xyphosphinyl) -L-leucyl-L-tryptophan × 2Na × 2H_2_O; PA) was purchased from PeptaNova GmbH (Sandhousen, Germany).

#### Preparation and Quality Control of ^99m^Tc-N_4_-GRP(14–27) and ^99m^Tc-N_4_-GRP(18–27)

Lyophilized N_4_-GRP(14–27) and N_4_-GRP(18–27) were dissolved in bidistilled water to a final 1 mM concentration and bulk solutions were distributed in 50 µL aliquots in Eppendorf vials (Protein LoBind Tube 1.5 mL; Eppendorf AG, Hamburg, Germany) and stored at −20 °C. For ^99m^Tc labeling, the following solutions were added into an Eppendorf tube containing 0.5 M phosphate buffer pH 11.5 (25 µL): 0.1 M sodium citrate (3 µL), [^99m^Tc]NaTcO_4_ (210 µL, 150–300 MBq) eluted from a commercial ^99^Mo/^99m^Tc generator (Ultratechnekow, Tyco Healthcare, Petten, The Netherlands), N_4_-GRP(14–27) or N_4_-GRP(18–27) stock solution (7.5 µL, 7.5 nmol), and finally, fresh SnCl_2_ solution in EtOH (5 µg, 5 µL). After 30 min incubation at ambient temperature the reaction mixture was neutralized by addition of 1 M HCl (4 µL) and EtOH was added (25 µL).

Quality control of the radiolabeled products comprised radioanalytical HPLC and instant thin-layer chromatography (ITLC). HPLC analyses were performed on a Waters Chromatograph coupled to a 2998 photodiode array UV detector (Waters, Vienna, Austria) and a Gabi gamma detector (Raytest RSM Analytische Instrumente GmbH, Germany). For analysis, a Waters Symmetry Shield RP-18 cartridge column (5 μm, 3.9 mm × 20 mm) was eluted at a 1.0 mL/min flow rate with the following gradient, 0% B to 40% B in 20 min, where A = 0.1% aq. trifluoroacetic acid (TFA), B = MeCN (System 1). Under these conditions ^99m^TcO_4_^−^ eluted at 1.8 min and ^99m^Tc-N_4_-GRP(14–27) and ^99m^Tc-N_4_-GRP(18–27) with a *t*_R_ > 12 min. For the detection of reduced hydrolyzed technetium (^99m^TcO_2_·× H_2_O) ITLC was conducted on ITLC-SG strips (Pall Corporation, New York, NY, USA), as previously described. The resultant ^99m^Tc-N_4_-GRP(14–27) and ^99m^Tc-N_4_-GRP(18–27) radioligands were used without further purification in all subsequent experiments.

Radioiodination of [Tyr^4^]BBN was performed using ^125^I ([^125^I]NaI in 0.1 N NaOH (pH 12–14) provided by MDS Nordion, Ottawa, ON, Canada) according to the chloramine-T methodology, as previously described [34]. Methionine was added to the purified radioligand solution to prevent reoxidation of Met^14^ to the corresponding sulfoxide and the resulting stock solution in 0.1% BSA-PBS was kept at −20 °C; aliquots thereof were used in competition binding assays (molar activity of 81.4 GBq/μmol).

### 4.2. In Vitro Assays

#### 4.2.1. Cell Lines and Culture

Human androgen-independent prostate adenocarcinoma PC-3 cells endogenously expressing the human GRPR (LGC Promochem, Teddington, UK) were used in the present study [43]. Cells were cultured in Roswell Park Memorial Institute (RPMI)-1640 medium, supplemented with 10% heat-inactivated fetal bovine serum (FBS), 100 U/mL penicillin, and 100 µg/mL streptomycin, and kept in a controlled humidified atmosphere containing 5% CO_2_ at 37 °C. Passages were performed weekly using a trypsin/EDTA (0.05%/0.02% *w*/*v*) solution. All culture media were purchased from Gibco BRL, Life Technologies and supplements were provided by Biochrom KG Seromed.

#### 4.2.2. Receptor Autoradiography

Binding affinities of N_4_-GRP(14–27) were determined by in vitro receptor autoradiography performed on cryostat sections of well characterized human tumor tissues, prostate carcinomas for GRPR, ileal carcinoids for NMBR and bronchial carcinoids for BB_3_R, as previously described [23]. The universal radioligand ^125^I-[DTyr^6^,*β*Ala^11^,Phe^13^,Nle^14^]BBN(6–14) (2 Ci/mol; ANAWA, Wangen Switzerland) was used as tracer known to identify all three bombesin receptor subtypes [6]. IC_50_ values are given in nM ± SEM.

#### 4.2.3. Competition Binding in PC-3 Cell-Membranes

Competition binding experiments against [^125^I-Tyr^4^] BBN were performed with N_4_-GRP(14–27) and N_4_-GRP(18–27), or with the unmodified parent peptide references GRP(14–27) and GRP(18–27) in PC-3 cell membranes. For the assay, triplicates per concentration point (concentration range: 10^−13^–10^−6^ M) of each test peptide were incubated together with the radioligand (~40,000 cpm per assay tube at a 50 pM concentration) in PC-3 cell-membrane homogenates in a total volume of 300 μL binding buffer (BB, 50 mM HEPES pH 7.4, 1% BSA, 5.5 mM MgCl_2_, 35 μM bacitracin) for 1 h at 22 °C in an Incubator-Orbital Shaker (MPM Instr. SrI, Bernareggio, Italy). Binding was interrupted by ice-cold washing buffer (WB, 10 mM HEPES pH 7.4, 150 mM NaCl) and rapid filtration (Whatman GF/B filters presoaked in BB) on a Brandel Cell Harvester (Adi Hassel Ing. Büro, Munich, Germany). Filters were washed with ice-cold WB and counted in an automatic well-type gamma counter (NaI(Tl) 3′-crystal, Cobra Packard Auto-Gamma 5000 series instrument). The IC_50_ values were calculated using nonlinear regression according to a one-site model applying the PRISM 2 program (Graph Pad Software, San Diego, CA, USA).

#### 4.2.4. Internalization Assay in PC-3 Cells

The internalization rates of ^99m^Tc-N_4_-GRP(14–27) and ^99m^Tc-N_4_-GRP(18–27) were compared in PC-3 cells. Briefly, PC-3 cells were seeded in six-well plates (~1 × 10^6^ cells per well) 24 h before the experiment. Approximately 50,000 cpm of either ^99m^Tc-N_4_-GRP(14–27) or ^99m^Tc-N_4_-GRP(18–27) (corresponding to 250 fmol total peptide in 150 μL of 0.5% BSA/PBS) was added alone (total) or in the presence of 1 μM [Tyr^4^]BBN (nonspecific). Cells were incubated at 37 °C for 15, 30, 60, and 120 min and incubation was interrupted each time by placing the plates on ice, removing the supernatants and rapid rinsing with ice-cold 0.5% BSA/PBS. Cells were then treated 2 × 5 min with acid wash buffer (2 × 0.6 mL, 50 mM glycine buffer pH 2.8, 0.1 M NaCl) at room temperature and supernatants were collected (membrane-bound fraction). After rinsing with 1 mL chilled 0.5% BSA/PBS, cells were lysed by treatment with 1 N NaOH (2 × 0.6 mL) and lysates were collected (internalized fraction). Sample radioactivity was measured in the γ-counter and percent internalized radioactivity was determined vs. total added activity. Results represent the average values ± sd of three experiments performed in triplicate.

### 4.3. Animal Studies

#### 4.3.1. In Vivo Stability Tests

For stability experiments, healthy male Swiss albino mice (30 ± 5 g, NCSR “Demokritos” Animal House Facility) were used. Test radioligand—^99m^Tc-N_4_-GRP(14–27) or ^99m^Tc-N_4_-GRP(18–27)—was injected as a 100 μL bolus (37–74 MBq, 3 nmol total peptide) in the tail vein together with injection solution (100 µL; control) or with a PA-solution (100 µL injection solution containing 300 µg PA). Animals were euthanized and blood (0.5–1 mL) was directly withdrawn from the heart in an ice-cold syringe and transferred in a prechilled EDTA and methionine-containing Eppendorf tube on ice. Blood samples were centrifuged for 10 min at 2000 g/4 °C and plasma was collected. After addition of an equal volume of ice-cold MeCN the mixture was centrifuged for 10 min at 15,000 g/4 °C. The supernatant was concentrated under a N_2_-flux at 60 °C to 0.05–0.1 mL, diluted with saline (0.4 mL), filtered through a 0.22 μm Millex GV filter (Millipore, Milford, CT, USA), and analyzed by RP-HPLC. The Symmetry Shield RP18 (5 μm, 3.9 mm × 20 mm) column was eluted at a flow rate of 1.0 mL/min with the following linear gradient (system 2): 0% B at 0 min to 10% B in 10 min and then in 40 min to 30% B; A = 0.1% aq. TFA and B = MeCN. The *t*_R_ of the intact radiopeptide was determined by coinjection with the ^99m^Tc-N_4_-GRP(14–27) and ^99m^Tc-N_4_-GRP(18–27) reference in the HPLC.

#### 4.3.2. Induction of PC-3 Xenografts in SCID Mice

A suspension containing freshly harvested human PC-3 cells (≈150 μL of a ≈1.2 × 10^7^ cells) was subcutaneously injected in the flanks of female SCID mice (15 ± 3 g, six weeks of age at the day of arrival, NCSR “Demokritos” Animal House Facility). The animals were kept under aseptic conditions and 4 weeks later developed well-palpable tumors (80–200 mg) at the inoculation sites.

#### 4.3.3. Biodistribution in PC-3 Xenograft-Bearing SCID Mice

For the biodistribution study, animals in groups of 4 received via the tail vein a 100 μL bolus of ^99m^Tc-N_4_-GRP(14–27) (180–370 kBq, corresponding to 10 pmol total peptide) coinjected either with injection solution (100 μL; control) or PA-solution (300 μg PA dissolved in 100 μL injection solution; 4 h + PA), or with excess [Tyr^4^]BBN (100 μL injection solution containing 50 μg [Tyr^4^]BBN for in vivo GRPR-blockade; 4 h block). Animals were euthanized at 1-, 4-, and 24-h pi; in the case of ^99m^Tc-N_4_-GRP(18–27) two animal groups were included at the 4-h pi interval, namely the control and PA groups described above. Mice were dissected; samples of blood, tumors, and organs of interest were collected, weighed, and measured for radioactivity in the gamma counter. Intestines and stomach were not emptied of their contents. Data was calculated as percent injected dose per gram tissue (%ID/g) with the aid of standard solutions and represent mean values ± sd, *n* = 4. All animal experiments were performed in compliance with national and European guidelines and approved by national authorities (Prefecture of Athens, EL 25 BIO 021; #1609 and #1610).

## Figures and Tables

**Table 1:** Affinities for the three human bombesin receptor subtypes.

Peptide Conjugate	IC_50_s in nM		
	GRPR ^1^	NMBR ^2^	BB_3_R ^3^
Universal ligand ^4^	1.5 ± 0.1 (3)	1.5 ± 0.2 (3)	3.5 ± 0.7 (3)
N_4_-GRP(14–27)	4.2 ± 1.0 (3)	72 ± 7.6 (3)	>1000 (3)
N_4_-GRP(18–27)	2.4 ± 1.0 (3)	106 ± 13 (2)	>1000 (3)
Demobesin 3	0.5 (2)	1.6 (2)	>100 (3)

The data represents the mean (±SEM; *n* = 3) for the cold universal ligand and for the two N_4_-GRP(14/18–27) analogs and the mean (*n* = 2) for Demobesin 3. ^125^I[DTyr^6^,*β*Ala^11^, Phe^13^,Nle^14^]BBN(6–14) was used as radioligand in all experiments; ^1^ expressed in human prostate cancer, ^2^ in human ileal carcinoids and ^3^ in human lung carcinoids, ^4^[DTyr^6^,*β*Ala^11^,Phe^13^,Nle^14^]BBN(6–14) was used as cold universal ligand.

**Table 2:** Biodistribution data for ^99m^Tc-N_4_-GRP(14–27), expressed as %ID/g mean ± sd, *n* = 4, in PC-3 xenograft-bearing SCID mice at 1-h, 4-h block, 4-h, and 24-h pi.

Tissue	1 h ^1^	4 h ^1^	24 h ^1^	4 h block ^2^
Blood	1.54 ± 0.17	0.10 ± 0.03	0.07 ± 0.01	0.08 ± 0.02
Liver	5.02 ± 0.46	3.75 ± 1.11	3.23 ± 0.78	6.12 ± 2.89
Heart	0.70 ± 0.07	0.11 ± 0.04	0.07 ± 0.01	0.28 ± 0.13
Kidneys	14.82 ± 2.22	4.83 ± 2.12	3.10 ± 0.73	4.85 ± 2.66
Stomach	1.02 ± 0.29	1.42 ± 0.93	0.78 ± 0.33	0.43 ± 0.18
Intestines	7.21 ± 0.52	7.61 ± 2.23	1.73 ± 0.23	1.45 ± 0.44
Muscle	0.29 ± 0.02	0.03 ± 0.01	0.06 ± 0.01	0.05 ± 0.02
Lungs	1.96 ± 0.33	0.49 ± 0.30	0.18 ± 0.04	0.65 ± 0.22
Pancreas	37.85 ± 1.95	35.24 ± 4.70	13.41 ± 0.77	0.83 ± 0.24
Tumor	10.20 ± 0.72	8.41 ± 4.16	4.50 ± 0.69	0.62 ± 0.24

^1^ Animal groups injected with 180–370 kBq/10 pmol peptide; ^2^ block mice group coinjected with 40 nmol [Tyr^4^] BBN for in vivo GRPR blockade.

**Table 3:** Comparative biodistribution data for ^99m^Tc-N_4_-GRP(14–27) and ^99m^Tc-N_4_-GRP(18–27), expressed as %ID/g mean ± sd, *n* = 4, in PC-3 xenograft-bearing SCID mice at 4-h pi (controls) and 4-h pi after coinjection of PA.

Tissue	^99m^Tc-N_4_-GRP(14–27)		^99m^Tc-N_4_-GRP(18–27)	
	4 h ^1^	4 h PA ^1,2^	4 h^1^	4 h PA ^1,2^
Blood	0.10 ± 0.03	0.23 ± 0.08	0.13 ± 0.04	0.21 ± 0.04
Liver	3.75 ± 1.11	4.99 ± 1.53	1.02 ± 0.17	2.13 ± 0.37
Heart	0.11 ± 0.04	0.89 ± 0.06	0.14 ± 0.10	0.47 ± 0.27
Kidneys	4.83 ± 2.12	11.68 ± 1.61	6.01 ± 1.38	7.66 ± 1.39
Stomach	1.42 ± 0.93	3.82 ± 1.10	1.12 ± 0.75	4.02 ± 0.66
Intestines	7.61 ± 2.23	21.35 ± 1.68	7.28 ± 0.60	16.23 ± 3.90
Muscle	0.03 ± 0.01	0.06 ± 0.02	0.03 ± 0.01	0.06 ± 0.01
Lungs	0.49 ± 0.30	1.06 ± 0.19	0.28 ± 0.09	0.84 ± 0.26
Pancreas	35.24 ± 4.70	110.32 ± 8.76	32.18 ± 5.91	95.39 ± 20.34
Tumor	8.41 ± 4.16	38.19 ± 4.79	7.08 ± 1.29	28.37 ± 8.05

^1^ Animal groups injected with 180–370 kBq/10 pmol peptide; ^2^ PA mice groups with animals coinjected with 300 µg PA to in situ inhibit NEP.
